# Longitudinal Analysis of Dysphagia and Factors Related to Postoperative Pneumonia in Patients Undergoing Esophagectomy for Esophageal Cancer

**DOI:** 10.1007/s00455-023-10618-6

**Published:** 2023-11-07

**Authors:** Asako Kaneoka, Haruhi Inokuchi, Rumi Ueha, Taku Sato, Takao Goto, Akihito Yamauchi, Yasuyuki Seto, Nobuhiko Haga

**Affiliations:** 1 The University of Tokyo Hospital Rehabilitation Center, 7-3-1 Hongo Bunkyo-Ku, Tokyo, 113-8655 Japan; 2 The University of Tokyo Hospital Swallowing Center, 7-3-1 Hongo Bunkyo-Ku, Tokyo, 113-8655 Japan; 3 Department of Otorhinolaryngology and Head and Neck Surgery, The University of Tokyo, 7-3-1 Hongo Bunkyo-Ku, Tokyo, 113-8655 Japan; 4 Department of Gastrointestinal Surgery, The University of Tokyo, 7-3-1 Hongo Bunkyo-Ku, Tokyo, 113-8655 Japan; 5 Rehabilitation Services Bureau, The National Rehabilitation Center for Persons With Disabilities, 4-1 Namiki, Tokorozawa, Saitama 359-8555 Japan

**Keywords:** Esophageal cancer, Dysphagia, MBSImP™, Kinematic analysis

## Abstract

Few studies have quantified longitudinal changes in swallowing in patients undergoing esophagectomy for esophageal cancer. This study longitudinally analyzed the changes in the Modified Barium Swallow Study Impairment Profile (MBSImP™) scores, swallowing kinematic measurements, and swallowing-related symptoms in patients undergoing esophagectomy. We also examined the association between identified swallowing impairment and aspiration pneumonia after surgery. We included consecutive patients who underwent esophagectomy and completed laryngoscopy and videofluoroscopy before, two weeks, and three months after surgery. We analyzed physiological impairments using the MBSImP. We also assessed the swallowing kinematics on a 5 mL thickened liquid bolus at three time points. Vocal fold mobility was assessed using a laryngoscope. Repeated measures were statistically examined for longitudinal changes in swallowing function. The association between the significant changes identified after esophagectomy and aspiration pneumonia was tested. Twenty-nine patients were included in this study. Preoperative swallowing function was intact in all participants. The timing of swallowing initiation and opening of the pharyngoesophageal segment remained unchanged after surgery. Tongue base retraction and pharyngeal constriction ratio worsened two weeks after surgery but returned to baseline levels three months after surgery. Three months after surgery, hyoid displacement and vocal fold immobility did not fully recover. Aspiration pneumonia occurred in nine patients after surgery and was associated with postoperative MBSImP pharyngeal residue scores. Decreased hyoid displacement and vocal fold immobility were observed postoperatively and persisted for a long time. The postoperative pharyngeal residue was associated with pneumonia and thus should be appropriately managed after surgery.

## Introduction

Esophageal cancer is the eighth most frequent cancer worldwide and the sixth leading cause of cancer-related deaths [1]. Radical resection of the esophagus with lymph node dissection has been the mainstay of treatment for locally advanced esophageal cancer [2]. This invasive treatment causes serious postoperative complications in nearly 60% of patients, including anastomotic leakage, strictures, and vocal fold immobility due to recurrent laryngeal nerve damage, pneumonia, and dysphagia [3]. Dysphagia and impaired swallowing function increase the risk of pneumonia [4] and mortality [5]. Thus, adequate management of dysphagia is crucial to achieve better health outcomes and quality of life after esophagectomy.

Clinically, dysphagic symptoms can be assessed using a videofluoroscopic swallowing study (VFSS) [6] and fiberoptic endoscopic examinations of swallowing (FEES) [7]. Previous research has reported aspiration in up to 49% [8–11] and residue in the vallecula and pyriform sinus in 45% and 80% of the patients postoperatively [12]. Furthermore, kinematic analyses of the postoperative VFSS [13, 14] demonstrated reduced displacement of the hyoid bone [8, 15, 16], reduced laryngeal elevation [9, 17], delayed swallowing reflex [8], and decreased opening of the upper esophageal sphincter [15]. Among these abnormalities, delayed swallowing reflex [8], reduced laryngeal elevation [8, 17], and vocal fold immobilit y [11, 18] are associated with postoperative aspiration.

Postoperative dysphagia has, however, been subject to several limitations in previous studies. First, VFSS was not performed preoperatively [12]. Thus, it is unclear whether the reported postoperative dysphagia was a newly developed complication of the surgical intervention or a worsening of the preexisting age-dependent decline in swallowing function, namely, presbyphagia [18, 19]. In addition, VFSS was not repeated in the long term. Thus, it is also unknown whether the reported swallowing impairment improves and if patients can return to a regular diet over time [18]. Second, most previous studies have reported swallowing symptoms, such as aspiration or pharyngeal residue only, and have not analyzed swallowing functions comprehensively using a standard tool, such as the Modified Barium Swallow Study Impairment Profile (MBSImP™) [6]. Furthermore, few studies have examined the relationship between pathological VFSS findings and postoperative aspiration pneumonia [9]. Thus, the impact of the identified swallowing abnormalities after esophagectomy on the development of pneumonia is uncertain.

This study longitudinally analyzed the changes in MBSImP scores, swallowing kinematic measurements, swallowing-related symptoms, and diet levels in patients undergoing esophagectomy. We then examined the association between the swallowing pathophysiology identified in the VFSS and aspiration pneumonia after esophagectomy.

## Methods

### Setting

This retrospective observational study was conducted at a teaching hospital in Tokyo, Japan. Written informed consent was obtained from all participants. The institutional review board approved the study protocol (Ref. No. 2018016NI).

### Participants

We used the data of consecutive patients who underwent curative esophagectomy for esophageal cancer between June 2017 and January 2020. We included patients who completed the laryngoscopic examination and VFSS at three time points: before, two weeks, and three months after surgery.

### VFSS Protocol

Otolaryngologists performed VFSS in the lateral projection with the patient upright at 1/30 s intervals before, two weeks after, and three months after the surgery. A hypoosmotic, nonionic, iodinated contrast agent (Iohexol; Omnipaque , Daiichi Sankyo Co., Ltd. Tokyo, Japan) was delivered via a syringe. A length reference marker (coin, 20.0 mm in diameter) was attached under each participant’s chin to calibrate the VFSS measurements. VFSS was performed using different volumes and consistencies of liquid contrast agents. According to the clinical decision of the examiner upon observing penetration-aspiration events, the evaluation of the different volumes and consistencies was discontinued to maximize patient safety and reduce the risk of adverse effects related to VFSS, since the materials could not be ejected by cough. VFSS recordings were archived as movie files in a hospital data storage system. In this study, we copied the movie files of the participants and de-identified them to allow subsequent analyses to be blinded. Although different volumes and consistencies of the test food were used for clinical VFSS, we only analyzed the data from the 5 mL of moderately thickened liquid swallows. The 5 mL moderately thickened liquid contrast agent (150–300 mPas) was swallowed in the head neutral position with no compensatory strategies [20] as all patients completed this trial. There was a considerable amount of missing data for thin liquids, following the decision to stop the trials for swallowing safety.

### Data Collection

#### Patient Characteristics

We captured the participants' demographics, disease status, and treatment characteristics through a chart review of the hospital’s electronic medical records. Preoperative nutritional status was determined using Onodera's prognostic nutritional index (PNI: 10 × albumin concentration [g/dL] + 0.005 × total lymphocyte count per mm^3^), which has been used to predict surgical outcomes of esophageal cancer [21]. Aspiration pneumonia was diagnosed by attending physicians based on a combination of infiltrates on chest radiography, clinical pneumonia symptoms, and laboratory criteria [22]. We analyzed the medical records during a follow-up period of three months after surgery to determine if aspiration pneumonia had occurred.

### Longitudinal Data

#### VFSS: The MBSImP

An MBSImP-certified speech-language therapist (SLT) scored the MBSImP to assess overall swallowing impairment. We excluded bolus preparation/mastication from the oral impairment (OI) components because we used only a liquid test bolus. The OI score was calculated as the sum of five of the six original components. We also excluded pharyngeal contractions from the Pharyngeal Impairment (PI) components because we did not perform the VFSS in the anteroposterior view in all patients. Thus, the PI score was the sum of nine of the ten original components.

#### VFSS: Swallowing Kinematic Measurements

We performed one temporal[13] and three displacement measurements[14] to analyze swallowing kinematics using a software application (Swallowtail, Belldev Medical, Arlington Heights, IL, USA). The following four measurements were collected:

- The period between the time when the bolus arrives at the pharyngoesophageal segment (BP1) and the first onset of hyoid movement leading to swallowing (H1) [13]
- The maximum displacement of the hyoid during swallowing (Hmax) [14]: The difference between the hyoid position at rest, when the bolus is in the anterior oral cavity and before any posterior movement of the bolus occurs, and the position where the hyoid is maximally elevated relative to the anterior corner of the second and fourth cervical vertebrae
- Maximum opening of the pharyngoesophageal segment (PESMax) [14]: The most significant expansion of the site designated as the PES (narrowest gap in the lateral view of the upper esophagus between C4 and C6) during swallowing
- Pharyngeal Constriction Ratio (PCR) [23]: The ratio of the area of the pharynx at rest and when it is maximally constricted.

#### VFSS: Swallowing Findings

Two assessors, the SLT and a physiatrist, independently judged the participants' levels of penetration/aspiration using the penetration-aspiration scale [24] (PAS score: 1: normal; 8: material enters the airway and passes below the vocal folds, and no effort is made to eject it). The raters were blinded to the participants' information and timing of the recorded VFSS assessment (before, two weeks after, or three months after surgery). Disagreements between the two assessors were resolved through discussion. The PAS scores were then categorized into three levels: regular (PAS score ≤ 2), penetration (3 ≤ PAS score ≤ 5), and aspiration (6 ≤ PAS score ≤ 8) [25]. The timing of penetration or aspiration was recorded before, during, and after swallowing [26]. We extracted the pharyngeal residue score from the MBSImP to determine the severity of pharyngeal residue. A score of 0 indicated “complete pharyngeal clearance,” and a score of 4 indicated “minimal to no pharyngeal clearance.”

#### Vocal Fold Immobility

We examined records of laryngoscopic examinations performed by otolaryngologists before, two weeks after, and three months after surgery. Vocal fold mobility was categorized as normal, unilateral, or bilateral.

#### Diet Levels

We examined the dietary levels of the participants before, two weeks after, and three months after surgery. Diet levels were graded using the ten-point Food Intake Level Scale (FILS; Level 1: no swallowing training except for oral care; Level 10: no dietary restriction, and the patient ingests three meals orally) [27]. The FILS scores were then categorized into three levels: no oral intake (FILS level ≤ 3), oral intake and alternative nutrition (4 ≤ FILS level ≤ 6), and oral intake alone (7 ≤ FILS level ≤ 10) [27].

### Statistical Analyses

Statistical analyses were performed using SAS (version 9.4; SAS Institute, Inc. Cary, NC, USA). Descriptive statistics were performed for continuous (means and standard deviations) and ordinal (medians and ranges) variables for patient demographic data. Categorical variables were presented as counts and percentages. The Friedman and post hoc Dunn–Bonferroni tests were performed to compare the ordinal repeated measurements across the three time points in the MBSImP, PAS, and FILS scores. The repeated measures of ANOVA with Bonferroni post hoc analyses were performed to analyze the longitudinal changes in VFSS kinematic measurements parametrically. The non-parametrical Wilcoxon’s rank sum test was performed to determine the association between postoperative aspiration pneumonia, pathological MBSImP scores, and swallowing kinematic measurements. The Fisher’s exact test was performed to determine the association between vocal fold immobility and aspiration pneumonia. A *p*-value of less than 0.05 was considered statistically significant.

## Results

### Participant Characteristics

Fifty-one patients underwent subtotal esophagectomy during the study period. Twenty-nine patients completed the laryngoscopic examination and the VFSS before, two weeks after, and three months after surgery and, thus, were included in this study. Twenty-two patients were excluded from this study as they did not complete the laryngoscopic examination and VFSS at the three time points. The majority of the study participants underwent subtotal esophagectomy, followed by reconstruction with a gastric tube conduit and cervical anastomosis. The posterior mediastinal route was usually used for the reconstruction. All the participants provided informed consent.

Table 1 displays the participant characteristics. The mean age of the participants was 64.4 ± 9.0 years. The mean preoperative PNI was 49.3 ± 5.4. Even though the procedures for subtotal esophagus resection varied, the sternohyoid and sternothyroid muscles were divided in all patients. The participants were nil per os for an average of 10.2 ± 7.0 days. Nine participants (31.0%) developed aspiration pneumonia after surgery. Pneumonia events occurred between 2 to 19 days postoperatively.

**Table 1:** Participant characteristics (*n* = 29)

Characteristics	Mean ± SD (Range)	*n* (%)
Age (years)	64.4 ± 9.0 (48.0–83.0)	
Gender		
Male		27 (93.0)
Female		2 (7.0)
Location of cancer		
Cervical		1 (3.5)
Thoracic		27 (93.0)
Abdominal		1 (3.5)
Histology		
Squamous cell carcinoma		28 (96.5)
Adenocarcinoma		1 (3.5)
Pathological stage		
I		5 (17.2)
II		12 (41.3)
III		11 (38.0)
IV		1 (3.5)
Preoperative prognostic nutritional index	49.3 ± 5.4 (39.0–59.0)	
Neoadjuvant chemotherapy		
Yes		22 (76.0)
No		7 (24.0)
Surgical procedure		
Thoracoscopy		20 (69.0)
Thoracotomy		9 (31.0)
Reconstruction route		
Retrosternal		2 (7.0)
Posterior mediastinal		27 (93.0)
Reconstruction organ		
Gastric		28 (96.5)
Colon		1 (3.5)
Anastomosis		
Cervical		27 (93.0)
Intrathoracic		2 (7.0)
Lymph node dissection		
Two-field		4 (14.0)
Three-field		25 (86.0)
Jejunostomy		
Yes		8 (27.6)
No		21 (72.4)
Tracheostomy		
Yes		0 (0)
No		29 (100)
Postoperative complications		
Anastomotic leakage		2 (7.0)
Vocal fold immobility		
None		2 (7.0)
Unilateral		20 (69.0)
Bilateral		7 (24.0)
Aspiration pneumonia		9 (31.0)

### Longitudinal Analyses

#### VFSS: The MBSImP

Table 2 shows the distribution (median and interquartile range) of the MBSImP scores for swallowing 5 mL of a thickened liquid of 29 participants at three time points: before, two weeks after, and three months after surgery. The global test demonstrated that the Oral Impairment Score was stable across the three time points (*p* = 0.28), and the Pharyngeal Impairment Score changed significantly over time (*p* <  0.0001). Table 3 shows the post hoc pairwise analyses of the MBSImP components with significance in the global test. The Pharyngeal Impairment Score decreased two weeks after surgery (*p* = 0.0002) but returned to the baseline level three months after surgery (*p* = 0.08). A similar trend was observed for tongue base retraction and pharyngeal residue.

**Table 2:** The MBSImP component scores before, two weeks after, and three months after esophagectomy (*n* = 29)

	Before surgery						2 weeks after surgery							3 months after surgery					Global statistics	
Score counts, %	0	1	2	3	4	Md (IQR)	0	1	2	3	4	Md (IQR)	0	1	2	3	4	Md (IQR)	χ^2^	Friedman *p*
Lip closure	28 96.6	1 3.4	0 0	0 0	0 0	0 (0)	29 100	0 0	0 0	0 0	0 0	0 (0)	28 96.6	1 3.4	0 0	0 0	0 0	0 (0)	1.00	0.61
Tongue control	29 100	0 0	0 0	0 0	–	0 (0)	29 100	0 0	0 0	0 0	–	0 (0)	27 93.1	2 6.9	0 0	0 0	–	0 (0)	4.00	0.14
Bolus transport	28 96.6	0 0	0 0	1 3.4	0 0	0 (0)	28 96.6	1 3.4	0 0	0 0	0 0	0 (0)	28 96.6	0 0	0 0	1 3.4	0 0	0 (0)	0.00	1.00
Oral residue	17 58.6	6 20.7	6 20.7	0 0	0 0	0 (1)	14 48.3	6 20.7	8 27.6	1 3.4	0 0	1 (2)	12 41.4	10 34.5	7 24.1	0 0	0 0	1 (1)	1.37	0.51
OI score						1 (2)						1 (2)						1 (2)	2.53	0.28
Initiation of swallow	20 69.0	8 27.6	1 3.4	0 0	0 0	0 (1)	15 51.7	10 34.5	1 3.4	2 6.9	1 3.4	0 (1)	19 65.5	8 27.6	2 6.9	0 0	0 0	0 (1)	2.68	0.26
Soft palate elevation	28 96.6	1 3.4	0 0	0 0	0 0	0 (0)	29 100	0 0	0 0	0 0	0 0	0 (0)	29 100	0 0	0 0	0 0	0 0	0 (0)	2.00	0.37
Laryngeal elevation	25 86.3	3 10.3	1 3.4	0 0	0 0	0 (0)	19 65.6	7 24.1	2 6.9	1 3.4	0 0	0 (1)	23 79.3	5 17.3	1 3.4	0 0	0 0	0 (0)	5.78	0.06
Anterior hyoid excursion	22 75.9	6 20.7	1 3.4	0 0	0 0	0 (0)	13 44.8	13 44.8	3 10.3	0 0	0 0	0 (1)	16 55.2	12 41.4	1 3.4	0 0	0 0	0 (1)	8.40	0.02
Epiglottic movement	28 96.6	1 3.4	0 0	0 0	0 0	0 (0)	23 79.3	2 6.9	4 13.8	0 0	0 0	0 (0)	24 82.8	3 10.3	2 6.9	0 0	0 0	0 (0)	5.26	0.07
LV closure	28 96.6	1 3.4	0 0	0 0	0 0	0 (0)	21 72.4	8 27.6	0 0	0 0	0 0	0 (1)	25 86.2	4 13.8	0 0	0 0	0 0	0 (0)	6.73	0.03
Pharyngeal stripping wave	26 89.7	2 6.9	1 3.4	0 0	0 0	0 (0)	21 72.4	8 27.6	0 0	0 0	0 0	0 (1)	23 79.3	5 17.3	1 3.4	0 0	0 0	0 (0)	3.45	0.18
PES opening	26 89.7	2 6.9	1 3.4	0 0	0 0	0 (0)	20 69.0	7 24.1	1 3.4	1 3.4	0 0	0 (1)	25 86.3	3 10.3	1 3.4	0 0	0 0	0 (0)	9.56	0.008
Tongue base retraction	22 75.9	7 24.1	0 0	0 0	0 0	0 (0)	12 41.4	7 24.1	6 20.7	4 13.8	0 0	1 (2)	15 51.7	8 27.6	4 13.8	2 6.9	0 0	0 (1)	18.1	0.0001
Pharyngeal residue	15 51.7	11 38.0	3 10.3	0 0	0 0	0 (1)	6 20.7	5 17.3	14 48.3	3 10.3	1 3.4	1 (2)	10 34.5	6 20.7	12 41.4	1 3.4	0 0	1 (2)	27.7	< .0001
PI Score						1 (3)						4 (6)						3 (3)	21.2	< .0001

*OI score* Oral Impairment Score, *PI score* Pharyngeal Impairment Score, *Md* median, *IQR* interquartile range, *LV closure* Laryngeal vestibule closure; *PES* Pharyngoesophageal Segment

**Table 3:** The Post-hoc pairwise analyses of MBSImP scores with significance in the global test (*n* = 29)

	Before vs. 2 weeks after surgery		Before vs. 3 months after surgery		2 weeks vs. 3 months after surgery	
	*z*-score	*p*-value	*z*-score	*p*-value	*z*-score	*p*-value
Pharyngeal Impairment Score	3.87	0.0002	2.03	0.08	1.84	0.07
Anterior hyoid excursion	1.77	0.23	1.18	0.71	0.59	> 0.99
Laryngeal vestibule closure	1.38	0.34	0.59	> 0.99	0.79	0.43
PES opening	1.38	0.34	0.20	> 0.99	1.19	0.24
Tongue base retraction	2.95	0.0063	1.58	0.23	1.38	0.17
Pharyngeal residue	3.81	0.0003	2.10	0.07	1.71	0.26

The *p*-values in the multiple comparisons were adjusted with the Dunn-Bonferroni approach

*PES* Pharyngoesophageal segment

#### VFSS: Swallowing Kinematic Measurements

Table 4 shows the changes in the kinematic measurements for a 5 mL thickened liquid swallowed by 29 participants at the three time points. The global test demonstrated that the maximum hyoid displacement (*p* < 0.0001) and PCR (*p* = 0.0068) significantly changed over time. Table 5 displays the post hoc pairwise analyses of the kinematic measurements. The timing measure (H1-BP1) and maximum PES opening remained stable. The PCR increased after surgery (*p* = 0.0016) and returned to preoperative levels after three months (*p* > 0.99). Hyoid displacement significantly decreased after surgery (*p* < 0.0001) and did not return to baseline after three months (*p *= 0.0016).

**Table 4:** The kinematic measurements before, two weeks after, and three months after esophagectomy (*n* = 29)

	Before surgery		2 weeks after surgery		3 months after surgery		Global statistics	
	Mean	SD	Mean	SD	Mean	SD	F-statistic	*p*-value
H1-BP1 (sec)	− 0.04	0.38	0.02	0.28	0.13	0.49	3.31	0.19
Hyoid displacement (cm)	1.84	0.38	1.31	0.56	1.35	0.64	19.01	< 0.0001
PES opening (cm)	0.85	0.25	0.91	0.34	0.84	0.26	1.45	0.48
PCR	0.10	0.12	0.20	0.16	0.11	0.12	9.98	0.0068

*SD* standard deviation; *H1-BP1* The duration (sec) between the time when the bolus arrives at the pharyngoesophageal segment (BP1) to the first onset of hyoid movement that leads to swallowing (H1); *PES* Pharyngoesophageal segment; *PCR* the pharyngeal constriction ratio

**Table 5:** Post-hoc pairwise analyses of kinematic measurements (*n* = 29)

	Before vs. 2 weeks after surgery		Before vs. 3 months after surgery		2 weeks vs. 3 months after surgery	
	*t*-score	*p*-value	*t*-score	*p*-value	*t*-score	*p*-value
H1-BP1	0.66	> 0.99	1.71	0.26	1.05	0.88
Hyoid displacement	4.27	< 0.0001	2.82	0.0016	1.44	0.45
PES opening	1.18	0.71	0.40	> 0.99	0.79	> 0.99
PCR	2.89	0.0116	0.46	> 0.99	2.43	0.05

The *p*-values in the multiple comparisons were adjusted with the Bonferroni approach

*H1-BP1* The duration (sec) between the time when the bolus arrives at the pharyngoesophageal segment (BP1) to the first onset of hyoid movement that leads to swallowing (H1); *PES* Pharyngoesophageal segment; *PCR* the pharyngeal constriction ratio

#### VFSS: Swallowing Findings

Table 6 shows the changes in swallowing findings regarding symptoms before and after surgery. None of the participants showed penetration or aspiration before surgery. Two of 29 participants (6.9%) showed penetration, and four of 29 participants (13.8%) demonstrated aspiration two weeks after surgery. Penetration remained in three participants (10.3%), and no participant presented with aspiration three months after surgery. As for the timing of airway invasion, all nine penetration and four aspiration events observed in the three time points occurred “during” swallowing. All the penetration and aspiration events occurred when the participants’ maximum laryngeal elevation seemed to be achieved. Table 7 shows the results of the pairwise analysis of the swallowing findings. No significant differences in the PAS were found across the groups.

**Table 6:** Changes in longitudinal data (*n* = 29)

Variables	Levels	Before surgery	2 weeks after surgery	3 months after surgery
		Counts (%)		
PAS	Normal	29 (100)	23 (79.3)	26 (89.7)
	Penetration	0 (0)	2 (6.9)	3 (10.3)
	Aspiration	0 (0)	4 (13.8)	0 (0)
Vocal fold immobility	None	29 (100)	2 (6.9)	13 (44.8)
	Unilateral	0 (0)	20 (69.0)	13 (44.8)
	Bilateral	0 (0)	7 (24.1)	3 (10.3)
FILS	No oral intake	0 (0)	5 (17.4)	0 (0)
	Oral intake and alternative nutrition	1 (3.4)	10 (34.5)	1 (3.4)
	Oral intake alone	28 (92.6)	14 (48.3)	28 (92.6)

*PAS* The penetration-aspiration scale, *FILS* food intake level scale

**Table 7:** Post-hoc pairwise analyses of longitudinal data

	Before vs. 2 weeks after surgery		Before vs. 3 months after surgery		2 weeks vs. 3 months after surgery	
	*z*-score	*p*-value	*z*-score	*p*-value	*z*-score	*p*-value
PAS	1.38	0.3360	0.39	> 0.99	0.98	0.3247
Vocal fold immobility	5.31	< 0.0001	3.15	0.003	2.17	0.03
FILS	2.823	0.0143	0.0656	> 0.99	2.89	0.0116

The *p*-values in the multiple comparisons were adjusted with the Dunn-Bonferroni approach

*PAS* The Penetration-aspiration scale, *FILS* Food Intake Level Scale

The pharyngeal residue in the MBSImP increased significantly two weeks after surgery (*p* = 0.0003) but returned to the baseline level three months after surgery (*p* = 0.07, Table 3).

#### Vocal Fold Immobility

All the participants showed normal vocal fold movements before surgery. Twenty of 29 participants (69.0%) presented with unilateral vocal fold immobility, and seven of 29 (24.1%) developed bilateral vocal fold immobility two weeks after surgery. Three months after surgery, symptoms remained unilaterally and bilaterally in 13 (44.8%) and 3 participants (10.3%), respectively (Table 6). Pairwise analysis indicated that vocal fold mobility significantly worsened two weeks after surgery (*p* < 0.0001; Table 7) and did not return to the baseline level three months after surgery (*p* = 0.003).

#### Diet Levels

Before surgery, 96.6% of participants were on a full oral diet. Of the participants, 48.3% achieved a full oral diet two weeks after surgery; all participants returned to a full oral diet three months after surgery (Table 6). The drop in diet levels was significant two weeks after surgery (*p* = 0.0143) but returned to baseline levels three months after surgery (*p* > 0.99; Table 7).

### Swallowing Pathophysiology and Postoperative Aspiration Pneumonia

Table 8 demonstrates the association between pneumonia status and the significant declines in MBSImP or kinematic measurements two weeks after surgery. Increased pharyngeal residue in the MBSImP group after surgery was associated with postoperative aspiration pneumonia (*p* = 0.05), while postoperative vocal fold immobility was not associated with aspiration pneumonia (*p* = 0.18).

**Table 8:** The association between the significant postoperative decline and aspiration pneumonia

Significant decline two weeks after surgery	Aspiration pneumonia		
	Absent (*n* = 20)	Present (*n* = 9)	*p*-value
	Median (IQR)		
MBSImP scores			
Pharyngeal Impairment	4.0 (1.0–7.5)	4.0 (3.0–8.0)	0.53
Tongue base retraction	1.0 (0.0–2.0)	0.5 (0.0–2.0)	0.75
Pharyngeal residue	1.5 (0.0–2.0)	2.0 (2.0–2.0)	0.05
Kinematic measurements			
Hyoid displacement (cm)	1.58 (1.28–1.98)	1.08 (0.88–1.62)	0.18
PCR	0.15 (0.07–0.26)	0.26 (0.11–0.31)	0.26

*IQR* Inter-quartile range, *PCR* the pharyngeal constriction ratio

## Discussion

This study delineated the longitudinal changes in swallowing physiology, dysphagic symptoms, and diet levels in patients undergoing esophagectomy. This study identified an association between pharyngeal residue and aspiration pneumonia after esophagectomy.

First, our preoperative VFSS assessment revealed that the swallowing functions of patients with esophageal cancer were intact before esophagectomy despite the assumption that presbyphagia [19] or sarcopenic dysphagia [28–30] may already exist owing to advanced age or malnutrition in patients with esophageal cancer preoperatively [18]. The study participants were younger (mean age = 64.4 years) than those with presbyphagia, which typically develops after 80 years of age [31]. In addition, they had a better nutritional status (mean PNI 49.3) than patients with sarcopenia preoperatively (mean PNI 46.0) [31]. Thus, preoperatively older or less-nourished patients may still be at risk of symptomatic or asymptomatic swallowing deficits, which could be exacerbated after surgery. Therefore, preoperative VFSS assessment remains valuable for identifying patients with baseline dysphagia and elevated risk of postoperative dysphagia.

Second, our longitudinal assessment demonstrated recovery of swallowing impairments after esophagectomy. Initiation of swallowing and PES opening were unchanged after surgery. Tongue base retraction and PCR worsened two weeks after surgery but returned to baseline levels three months after surgery. However, once impaired after surgery, hyoid displacement during swallowing and vocal fold immobility did not fully recover after three months.

The temporary decline in tongue base retraction and pharyngeal squeezing indicated by PCR were unexpected changes after surgery, as the surgical procedure involved in esophagectomy or lymphadenectomy does not typically affect these swallowing mechanisms. However, as previously reported, it is possible that exposure to physical stress during invasive surgery and prolonged postoperative nil per os status overwhelmed the participants' functional reserves, causing temporal weakness in the lingual or pharyngeal muscles [19]. Consequently, bolus transport was tentatively affected, increasing pharyngeal residue after esophagectomy. Another potential reason for the tentative impairment in pharyngeal squeezing is iatrogenic damage to the ansa cervicalis and nerve rootlets during neck dissection, although the impact of neck dissection on pharyngeal movement was likely limited [32, 33].

Contrary to the decline in tongue base retraction and PCR, the reduced hyoid displacement during swallowing and vocal fold immobility is likely due to the direct impact of surgical procedures on swallowing mechanisms and, therefore, is persistent. In cervical lymph node dissection during esophagectomy, the infrahyoid muscles are either retracted or partially divided [34]. The damaged infrahyoid muscles become scarred, thereby hindering the lifting of the hyolaryngeal complex [9]. Scar formation around the trachea may also produce a counterforce against the hyolaryngeal elevation [17]. Furthermore, damage to the recurrent laryngeal nerve during lymphadenectomy increases the risk of vocal fold immobility after esophagectomy [35]. All the study participants underwent lymphadenectomy, which may be a reason for the higher incidence of vocal fold immobility observed in our study compared to that in previous reports in which esophagectomy was performed without lymphadenectomy (12.7% [8], 14.9% [11]). These surgery-induced changes might have allowed postoperative airway invasion in the participants. As previously reported, reduced hyolaryngeal elevation [8, 17] and vocal fold immobility [11, 18] were associated with aspiration after esophagectomy.

Despite persistent deficits in the movement of the hyoid and vocal folds, no aspiration events occurred three months after surgery. One possible reason for this favorable outcome is that the participants may have acquired maneuvers for safe swallowing, such as super supraglottic swallow [36] or chin-tuck swallow [37]. Kumai et al. reported that patients who underwent esophagectomy with three-field lymph node dissection had already acquired a chin-down swallow at postoperative VFSS without specific guidance and thus showed lower PAS scores than those who did not [38]. Our longitudinal research added that the compromised airway protective mechanism could continue for up to three months, emphasizing the importance of postoperative education for patients with compensatory strategies for airway protection.

Finally, our study found that pharyngeal residue, among other swallowing-related abnormalities, identified through a thorough swallowing assessment, is the single postoperative change associated with aspiration pneumonia. The pharyngeal residue, specifically the residue in the vallecula, is related to post-swallow aspiration [39]. However, in our study, all penetration and aspiration events were observed during swallowing when the participants’ maximum laryngeal elevation seemed to be achieved; these were probably due to incomplete airway closure. No aspiration of post-swallow residue was observed during VFSS, which might be due to the limitation of the test bolus volume to 5 mL. As such, accumulated residue with larger bolus in real-life meals can result in aspiration, thereby leading to aspiration pneumonia. Therefore, managing pharyngeal residues is essential to decrease penetration and aspiration after esophagectomy.

The study did not specifically analyze the location of the residue. However, the significant postoperative impairments identified in this study are known contributors to pharyngeal residue [40]: reduced tongue base retraction, reduced pharyngeal contraction, and decreased hyoid displacement. This study could not examine the longitudinal changes in several other factors, such as hyolaryngeal approximation or pharyngeal shortening during swallowing, which might affect postoperative residue. Further kinematic analyses may reveal the detailed contributions of swallowing mechanisms to pharyngeal residue, which may, in turn, help identify potential targets for postoperative swallowing exercises.

The current study has several limitations, similar to other retrospective observational studies [41]. First, the results were based on a limited sample size from a single-center setting, hampering generalizability and limited univariate statistical analyses. For example, most participants had thoracic esophagus cancers. Other participant characteristics, such as pathological stage, surgical procedure, or reconstruction routes, may have influenced postoperative swallowing impairments. To our knowledge, no other comprehensive longitudinal analyses of the kinematics and dysphagic symptoms have been published. Therefore, our study provides unique findings regarding dysphagia associated with esophagectomy. Second, our inclusion criteria required three-time VFSS data, potentially limiting patients with severe medical conditions and prolonged serious dysphagia who could not undergo VFSS from participating in the study. Third, the analysis was restricted to swallowing of only 5 mL of thickened liquid in this study due to missing data regarding thin liquid trials resulting from VFSS discontinuation for swallowing safety. Thin-liquid swallowing trials might have revealed more penetration and aspiration events, leading to additional findings of dysphagia after esophagectomy. Similarly, decreased UES openings may be observed for larger bolus volumes, as UES opening is volume dependent [14]. However, as this study was performed postoperatively in an acute hospital setting, swallowing safety during the VFSS was prioritized. Finally, observer bias may be involved in assessing the MBSImP since only one SLP served as a rater. However, to minimize bias, we ensured that the scoring was performed by an MBSImP-certified rater. We also blinded the two raters when assessing the PAS.

Future large-scale studies are warranted to examine longitudinal changes in swallowing function, kinematics, and symptoms of other dysphagia-related variables. Studies should also investigate factors associated with patients with severe dysphagia using various test boluses after esophagectomy.

## Conclusion

This study demonstrated the longitudinal changes in swallowing physiology using VFSS and diet levels in patients undergoing esophagectomy. Preoperative swallowing function was intact, while postoperative vocal fold immobility, MBSImP parameters (tongue base retraction and pharyngeal residue), and kinematic measurements (hyoid displacement and PCR) worsened postoperatively. Of these changes, reduced hyoid displacement and vocal fold immobility remained after three months; however, no aspiration events occurred, and all patients returned to a regular diet. Postoperative pneumonia was associated with pharyngeal residue, highlighting the importance of postoperative education to improve swallowing safety and efficiency.
